# Extent of exposure to scented candles and prevalence of respiratory and non-respiratory symptoms amongst young university students

**DOI:** 10.1186/s12889-023-15001-6

**Published:** 2023-01-11

**Authors:** Noor Al Khathlan, Meaad Basuwaidan, Sarah Al Yami, Fatimah Al-Saif, Salam Al-Fareed, Khalid Ansari

**Affiliations:** Department of Respiratory Care, College of Applied Medical Sciences, Imam Abdulrahman Bin Faisal University, Dammam, Saudi Arabia

**Keywords:** Cough, Headache, Health-related problems, Prevalence, Scented candle

## Abstract

**Background:**

Incense burning such as scented candles are commonly used in Arabian Gulf regions as it is thought to produce relaxing effects on people’s mood. This study is conducted to examine the prevalence of scented candles’ usage, extent of exposure and its effects on individuals’ health based on symptoms prevalence in young university students.

**Material and method:**

A cross-sectional study was conducted on university students from different regions in Saudi Arabia. Data was collected in March 2020 using an online questionnaire survey adapted from The European Community Respiratory Health Survey-II (ECRHS-II). Inclusion criterion for recruitment was students with non-smoking status. Descriptive statistics were used to report demographic data on the extent of exposure to scented candles (in terms of frequency and duration) and the presence of symptoms. Multiple logistic regression analysis was used to assess the relationship between scented candles exposure and respiratory and other health-related problems.

**Results:**

The prevalence of scented candles usage was 65.7% (472/718) among the respondents. However, its pervasiveness was significantly higher in females than in male respondents (74.9% vs. 28.4%; *p* = 0.0001). Among the scented candle users, 34.8% of the respondents used the scented candles more than 4 times a month and 40.2% of the respondents lit the scented candles for 20–40 min. A total of 117 (24.8%) respondents reported health-related problem and the top three health problems were headache 72 (15.2%), shortness of breath 42 (8.9%) and cough 37 (7.8%). The scented candle usage 5–6 times a week showed significantly lower wheezing (OR = 0.10, 95%CI 0.02–0.54, *p* = 0.008). The duration of more than 60 min of scented candle exposure showed higher occurrence of headache 1.42 times (95% CI = 0.68–2.96), sneezing 1.29 times (95% CI = 0.42-4.00) and wheezing 1.23 times (95% CI = 0.48–3.13), though the association was not significant.

**Conclusion:**

The results show that scented candle usage is more prevalent among female university students in Saudi Arabia. The common health-related problems associated with scented candle exposure were headache, shortness of breath and coughing.

## Introduction

The use of fragrance products such as Oud and scented candles are the most popular, affordable, and readily accessible luxury home items that infuse homes with a sensation of warmth and special ambiance in Saudi Arabia [1]. The aroma from a lit scented candle is released through the evaporation of the fragrance from the hot wax pool and from the solid candle itself releasing volatile organic compounds (VOCs). Even the fragranced products labeled as green or organic, can also emit surreptitious hazardous air pollutants [2]. The burning of paraffin releases VOCs into the air like acetone, benzene, and toluene [3].

The synthetic fragrances that are used in making candle scents usually contain phthalates. As the candles burn, phthalates are released into the air and can be inhaled or absorbed through the skin. When the phthalates enter the bloodstream, they can exacerbate allergic symptoms and asthma and alter hormone levels [4]. A number of studies have reported that most fragrances products like scented candles, cleaning products, deodorants, scents of laundry products, air-fresheners are associated with generalized headaches, coughing, shortness of breath, migraine headache, asthma attacks, dermatitis or allergic rhinitis [5–11]. The U.S. public health authorities have reported that a regular burning of several scented candles indoors can expose the resident to detrimental quantities of organic chemicals [12]. Breathing of VOCs is linked to irritation in the eyes, nose and throat, exertion in breathing and nausea, and can also result in damaging the central nervous system and other organs [13].

Moreover, the burning of scented candles also results in release of particulates matter (PM), which is one of the contributing factors for the development of adverse health effects and indoor air pollution [14]. According to Danish CISBO project report an exposure to high levels of PM may have negative affect on the pulmonary and the cardiovascular system [15]. The PM released from candles is very small and when these particles enter the respiratory system, they get deposited in the alveoli [15]. The insoluble parts of the PM are eliminated slowly from system and possibly the accumulation of these particles in the alveoli may be responsible for respiratory and cardiovascular problems.

Saudi Arabia and other Arab countries are regions where the use of scented candles domestically and in social gathering is very popular [16]. The higher usage of the scented candles in this region reflects a lack of awareness and knowledge about high emissions of VOCs and fine particles from burning candles and its significant association with health risk among the people who use a lot of candles. Moreover, there is a gap regarding the extent of the usage of scented candles and its effect on health from Arab countries. Therefore, the present study was designed to examine the prevalence of scented candles’ usage, extent of exposure and its effects on individuals’ health based on the prevalence of symptoms among the young university students.

## Materials & methods

A cross-sectional survey was conducted in all regions (Eastern, Western, Northern, Southern and Central) of Saudi Arabia from March-June, 2020. The ethical approval was obtained from Imam Abdulrahman Bin Faisal University Institutional Review Board (IRB). The inclusion criterion for the study was non-smoking university students. The exclusion criterion was current or ex-smokers. The data was collected through an online survey, which was disseminated amongst study participants through social media (WhatsApp and Twitter). The survey was adopted from The European Community Respiratory Health Survey II (ECRHS II) [11]. The survey was translated into Arabic language as the participants were mostly Arabic speakers. Three experts validated the Arabic version of the questionnaire. Moreover, the survey circulated among the participants had both the original English version and the translated Arabic version for clarity. The questionnaire consisted of three major parts: (1) participant’s demographic information; (2) questions regarding participant’s personal exposure to fragranced candles, the duration of exposure, and any related health problems and (3) questions related to respiratory symptoms. The demographic data were collected from all the participants, while extent of usage of scented candle and respiratory symptoms were collected only from the participants (*n* = 472) who used scented candles.

### Statistical analysis

Data was collected and analyzed using the IBM Statistical Package of Social Science (SPSS), Versions 23 Armonk, New York. Descriptive statistics was used to report demographic data (age, gender, nationality, economic status, area of residency) of the participants, the extent of exposure to scented candles, and frequency of its use. Chi-Square test was used to study the relationship between the demographics of participants and usage of scented candles, exposure to a scented candle and the respiratory related symptoms. Multiple logistic regression analysis was used to assess the association of frequency and duration of scented candles use and symptoms.

## Results

**Fig. 1:**
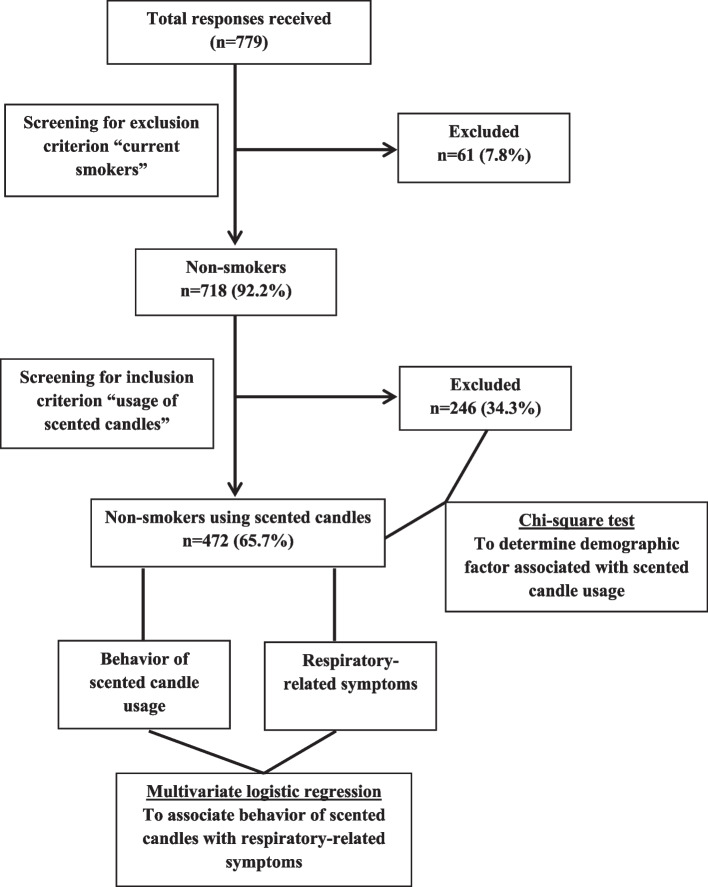
Flow chart depicting the selection of participant responses for analysis

Out of total 779 responses, 61 respondents did not meet the inclusion criteria and were excluded. The distribution and selection of participants is presented in Fig. 1.

### Demographic factors associated with scented candle usage

Among young university students, the scented candle usage prevalence is 65.7%. It was observed that a significantly higher percentage of female respondents [432 (90.5%)], medium economic status respondents [394 (83.5%)] and Eastern region respondents [357 (75.6%)] reported usage of scented candles, while a significantly lower percentage of low economic status respondents and Central [50 (10.6%)] and Southern [4 (0.8%)] regions respondents reported usage of scented candles. Age and nationality of the respondents showed no significant association with the usage of scented candles (Table 1).

**Table 1:** Socio-demographic characteristics of the study population

Socio-demographic characteristics	Total participants (*n*=718)		Chi-square	***p***-value
	Use scented candles (*n*=472)	Not use scented candles (*n*=246)		
**Age (years)**				
17-21	205 (43.4%)	114 (46.3%)	χ2(3) = 2.922	0.404
22-26	177 (37.5%)	92 (37.4%)		
27-31	34 (7.2%)	10 (4.1%)		
>31	56 (11.9%)	30 (12.2%)		
**Gender**				
Female	432 (91.5%)	145 (58.9%)	χ2(1) = 108.78	0.0001*
Male	40 (8.5%)	101 (41.1%)		
**Nationality**				
Saudi	458 (97.0%)	233 (94.7%)	χ2(1) = 2.40	0.121
Non-Saudi	14 (3.0%)	13 (5.3%)		
**Area of Residency**				
East	357 (75.6%)	149 (60.6%)	χ2(1) = 17.64	0.0001*
West	56 (11.9%)	32 (13.0%)	χ2(1) = 0.197	0.657
Central	50 (10.6%)	39 (15.9%)	χ2(1) = 4.12	0.042*
South	4 (0.8%)	23 (9.3%)	χ2(1) = 32.30	0.0001*
North	5 (1.1%)	3 (1.2%)	χ2(1) = 0.038	0.846
**Economic Status**				
Low	41 (8.7%)	42 (17.1%)	χ2(1) = 11.12	0.0008*
Medium	394 (83.5%)	175 (71.1%)	χ2(1) = 14.96	0.0001*
High	37 (7.8%)	29 (11.8%)	χ2(1) = 3.02	0.082

* *p*>-value significant

### Exposure to scented candle in the last 12 months in terms of frequency and duration

Most of the respondents 164 (34.8%) reported use of scented candles more than 4 times a month and among these 164 respondents only 26 (15.9%) participants used it daily. The majority of respondents 322 (68.2%) used the scented candles once or twice in a single day and 190 (40.2%) respondents reported lighting the scented candles for 20–40 min (Table 2)

**Table 2:** Extent of exposure to scented candles among study participants (*n*=472)

Extent of exposure to scented candles in the last 12 months?	n (%)
**How often do you use scented candles in a month**	
Once	130 (27.5)
Twice	92 (19.5)
3 Times	86 (18.2)
4 Times or more	164 (34.8)
**If more than 4 time (in month) how many times you use it in a week**	
1-2 Times	60 (36.6)
3-4 Times	63 (38.4)
5-6 Times	15 (9.1)
Daily	26 (15.9)
**How many times you use it in a single day**	
1-2 Times	322 (68.2)
3 Times or more	150 (31.8)
**How long you keep the candle lit for each time**	
< 20 minutes	136 (28.8)
20-40 minutes	190 (40.2)
40-60 minutes	81 (17.2)
> 60 minutes	65 (13.8)

### Prevalence of symptoms and other health conditions in the last 12 months among Scented candle users

A total of 117 (24.8%) respondents reported symptoms using ECRHS. The most common symptoms were headache (72) (15.2%), shortness of breath (42) (8.9%) and cough (37) (7.8%) (Table 3). Amongst the 472 respondents who used scented candles, 77 (16.3%) respondents reported to have history of diagnosed respiratory illness in the past 12 months. The most common respiratory-related health problem reported in the past 12 months were waking up with feeling of tightness in chest (36%) and waking up due to attack of coughing (33.1%) (Table 4).

**Table 3:** Prevalence of respiratory and non-respiratory symptoms among scented candle users (*n*=472)

Types of health problems amongst participants in the last 12 months?	n (%)
Headache	72 (15.2)
Asthma attack	4 (0.8)
Sinusitis	13 (2.7)
Cough	37 (7.8)
Shortness of breath	42 (8.9)
Skin irritation	6 (1.3)
Other	17 (3.6)
Only one symptom	73 (15.5)
Two symptoms	32 (6.8)
More than 2 symptoms	12 (2.5)
Total participants reporting adverse health problems	117 (24.8%)

**Table 4:** Prevalence of respiratory-related health problems among scented candles user (*n*=472)

Respiratory-related health problems	n (%)
History of diagnosed respiratory illness at any time in the last 12 months?	77 (16.3)
Have you had wheezing or whistling in your chest at any time in the last 12 months?	69 (14.6)
Have you woken up with a feeling of tightness in your chest at any time in the last 12 months?	170 (36)
Have you been woken by an attack of shortness of breath at any time in the last 12 months?	81 (17.2)
Have you been woken by an attack of coughing at any time in the last 12 months?	156 (33.1)
Have you had an attack of asthma in the last 12 months?	45 (9.5)
Are you currently taking any medicine (including inhalers, aerosols or tablets) for asthma	39 (8.3)
Nasal allergies including hay fever	118 (25)

### Association between frequency and duration of scented candle use and reported symptoms

Table 5 shows the multi-nominal logistic regression analysis results of association between extent of scented candles usage and reported symptoms. The usage of scented candles 4 times or more in a month, showed to increase the occurrence of chest tightness 1.27 times (95% CI = 0.72–2.23), shortness of breath 1.51 times (95% CI = 0.72–3.18), and nasal allergy 1.27 times (95% CI = 0.71–2.27), though the association was not statistically significant (*p* > 0.05). Similarly, the use of scented candles daily showed to increase occurrence of sneezing 1.41 times (95% CI = 0.13–15.38) and cough 1.84 times (95% CI = 0.57–5.90) (*p* > 0.05), while scented candle usage 5–6 times a week showed significantly lower wheezing (OR = 0.10, 95%CI 0.02–0.54, *p* = 0.008). Furthermore, three or more times usage of scented candle in a day showed to increase occurrence of shortness of breath 1.72 times (95% CI = 0.89–3.33) and duration of more than 60 min of scented candle exposure showed higher occurrence of headache 1.42 times (95% CI = 0.68–2.96), sneezing 1.29 times (95% CI = 0.42-4.00) and wheezing 1.23 times (95% CI = 0.48–3.13), though the association was not significant.

**Table 5:** Multi-nominal logistic regression analysis of association between frequency and duration of scented candle use and reported symptoms (*n*=472)

Extent of exposure to scented candles	Reported symptoms [OR (95% CI); ***p***-value]						
	Headache	Sneezing	Wheezing	Cough	Chest tightness	Shortness of breath	Nasal allergies
Frequency of use in a month							
Once	Ref	Ref	Ref	Ref	Ref	Ref	Ref
Twice	1.02 [0.51-2.02] 0.952	0.55 [0.20-1.50] 0.243	0.57 [0.25-1.33] 0.193	1.05 [0.57-1.94] 0.877	1.03 [0.54-1.98] 0.925	1.20 [0.53-2.73] 0.662	1.03 [0.54-1.98] 0.929
3 Times	0.66 [0.34-1.31] 0.238	0.47 [0.17-1.32] 0.151	0.65 [0.27-1.56] 0.337	1.13 [0.60-2.14] 0.705	1.71 [0.85-3.44] 0.133	0.99 [0.42-2.31] 0.981	1.03 [0.53-2.01] 0.932
4 Times or more	0.65 [0.36-1.16] 0.145	1.04 [0.38-2.85] 0.942	0.74 [0.34-1.63] 0.457	1.07 [0.62-1.82] 0.814	1.27 [0.72-2.23] 0.406	1.51 [0.72-3.18] 0.281	1.27 [0.71-2.27] 0.418
Frequency of use in a week							
1-2 Times	Ref	Ref	Ref	Ref	Ref	Ref	Ref
3-4 Times	0.51 [0.20-1.33] 0.169	0.93 [0.19-4.58] 0.926	0.62 [0.16-2.34] 0.477	1.03 [0.44-2.43] 0.939	0.80 [0.34-1.90] 0.617	1.14 [0.33-3.93] 0.838	1.00 [0.39-2.53] 0.997
5-6 Times	0.78 [0.16-3.74] 0.758	0.36 [0.04-2.89] 0.337	0.10 [0.02-0.54] 0.008*	4.19 [0.78-22.61] 0.095	0.87 [0.20-3.72] 0.854	1.16 [0.14-9.27] 0.892	0.86 [0.18-4.01] 0.848
Daily	0.55 [0.16-1.87] 0.343	1.41 [0.13-15.38] 0.729	0.44 [0.09-2.13] 0.305	1.84 [0.57-5.90] 0.305	1.00 [0.32-3.16] 0.999	0.37 [0.09-1.49] 0.163	1.03 [0.31-3.45] 0.960
Frequency of use in a day							
1-2 Times	Ref	Ref	Ref	Ref	Ref	Ref	Ref
3 Times or more	0.81 [0.50-1.31] 0.396	0.97 [0.44-2.14] 0.949	0.95 [0.51-1.79] 0.878	1.03 [0.66-1.63] 0.883	1.13 [0.70-1.84] 0.612	1.72 [0.89-3.33] 0.107	1.12 [0.69-1.83] 0.642
Duration of exposure							
< 20 minutes	Ref	Ref	Ref	Ref	Ref	Ref	Ref
20-60 minutes	1.11 [0.67-1.83] 0.694	0.92 [0.41-2.08] 0.835	0.92 [0.48-1.79] 0.817	1.10 [0.68-1.77] 0.706	1.16 [0.70-1.93] 0.563	0.83 [0.42-1.63] 0.584	0.94 [0.56-1.57] 0.811
> 60 minutes	1.42 [0.68-2.96] 0.349	1.29 [0.42-4.00] 0.654	1.23 [0.48-3.13] 0.669	0.72 [0.37-1.40] 0.331	0.84 [0.41-1.73] 0.645	0.50 [0.21-1.21] 0.125	0.80 [0.39-1.65] 0.551

## Discussion

To our knowledge, this is the first study from Saudi Arabia to look for frequency of scented candle usage and its association with health problems. This study provides the insight on the prevalence of scented candle usage among young Saudi university student and also examined the extent of scented candle usage that could be responsible to cause adverse health effects in young Saudi university students.

This study found an overall of 65.7% of university students use scented candles. However, a general population surveyed in America reported the prevalence of using fragranced household products which included scented candle as 77%, which is higher than present study. This difference may be because the prevalence included other fragranced products like toilet paper, trash bags, baby products and also it may be due to the differences in demographic, social and cultural profiles [17]. Moreover, the prevalence of fragranced products were reported that both males and females were equally exposed, while in the present study we found higher prevalence of scented candle usage among female university students [17]. The exposure to some phthalate metabolites are found to have endocrine-disrupting effects like increased estradiol and decreased testosterone levels [18]. In adult males high levels of urinary phthalate metabolites are reported to decrease the semen quality and in females association of DEHP with endometriosis have been reported [19–21]. Moreover, studies have reported association of DEHP with increased pregnancy loss; however there are conflicting reports also [22–25]. A study has reported that increased MEP exposure results in decreased probability of succeeding a pregnancy within six months of trying conception [26]. The high percentage of scented candles usage amongst Saudi youth highlights an alarming fact of their higher exposure to the VOCs [2, 3, 14] that affect their health adversely.

The adverse symptoms prevalence is 117 (24.8%) among the university students of Saudi Arabia who used scented candles, which was comparatively higher than the results reported by Steinemann, 2019 in the general population of United States (22.3%), Australia (20.3%), United Kingdom (13.9%) and Sweden (17.9%) for the other types of fragranced product which included scented candles [27]. This finding suggests that the emission of VOCs, phthalates and PM by burning of scented candles like other fragrances products may be a reason for development of these adverse symptoms.

In the present study, the non-respiratory symptoms observed in the respondents was headache (15.2%), the prevalence of which was similar to that reported by Steinemann, 2019 in US (15.7%) and Sweden (16.1%) but higher than Australia (10%) and UK (8.4%) [27]. In the present study, the respiratory-related symptoms of cough and shortness of breath was present in 16.7% respondents, which was in concordance with the findings from Australia (16.7%) but comparatively lower than US (18.6%) and Sweden (20%) [27].

Similarly in the present study skin irritation was reported by 1.3% respondents, which was comparatively lower than the four countries studied by Steinemann ranging from 6.5 to 10.6% [27]. The prevalence of asthma attack are consistent with previous studies [16, 27] but were relatively lower at 0.8% than in US (8%), Australia (7.6%), UK (6.8%) and Sweden (5.5%) [27]. This difference in prevalence may be due to the differences in sample size and age range. Also, the prevalence of asthma in general is higher in European countries than in the Asian population [28, 29]. A study also reported that individuals with asthma and chemical sensitivity showed adverse effects to scented products, air fresheners, and scented laundry products in higher proportions than the general public [10]. Another study conducted on workers in the State of California showed that 3.8% confirmed work related asthma cases reported from 1993 to 2012 were linked to the exposure to fragranced products [30].

However, the comparison with other studies does not provide a clear insight on the health-related problems as the studies differed in characteristics of study population and selection of fragrance product but also in terms of sample size, regional demographics and inclusion criteria. In the present study, only wheezing showed significant association with the exposure to scented candle usage among Saudi university students and health-related problems, which may be due to small sample size and selection criteria of study population. In the present study, participants were non-smokers and majority under the age of 30 years so it is expected that they had a normal respiratory function and highly active defense mechanism. However, the presence of asthma like symptoms in scented candle users in this study may lead to adult onset asthma later in life.

### Limitations

The study design used was cross-sectional, so both the cause (candle usage) and effect (respiratory and non-respiratory symptoms) data were collected simultaneously which might have led to recall bias. The survey did not include any specific questions related to the exact type and component of the scented candles used by the participants, so it might be possible that some participants used organic, toxic or non-toxic candles limiting or maximizing the severe effects of chemical based scented candles [31, 32]. The questionnaire did not collect the data on the pre-existing medical condition of the respondents and health-related problem data were not collected from the respondents who did not used scented candles. Moreover, the information about symptoms were self-reported, therefore it may result in under-estimated or over-estimated.

Further research studies are needed to be conducted with a more detailed list of fragrance products for better understanding of association between the fragrance use and health problems. A repeat survey of the respondents should be planned after a period of five to ten years if possible to evaluate the long term effect of scented candle usage on respiratory health.

## Conclusion

The finding of present study shows that scented candle use is common especially among female university students in Saudi Arabia. The health related problems were present in 24.8% of the respondents. The respondent’s exposure to scented candles for more than 60 min showed higher occurrence of headache, sneezing and wheezing.

## Data Availability

The datasets used and/or analysed during the present study will be made available from the corresponding author on reasonable request.
